# FUBP3 regulates chronic myeloid leukaemia progression through PRC2 complex regulated PAK1‐ERK signalling

**DOI:** 10.1111/jcmm.17584

**Published:** 2022-12-07

**Authors:** Mugdha Sharma, Seetharam Anandram, Cecil Ross, Sweta Srivastava

**Affiliations:** ^1^ Department of Medicine St. John's Medical College and Hospital Bengaluru India; ^2^ St. John's National Academy of Health Sciences Bengaluru India; ^3^ Department of Clinical Hematology St. John's Medical College and Hospital Bengaluru India; ^4^ Department of Transfusion Medicine and Immunohematology St. John's Medical College and Hospital Bengaluru India

**Keywords:** CML, derivative chromosome 9, ERK, fibrosis, FUBP3, PAK1, PRC2, Sokal, TKI

## Abstract

The development of resistance and heterogeneity in differential response towards tyrosine kinase inhibitors (TKI) in chronic myeloid leukaemia (CML) treatment has led to the exploration of factors independent of the Philadelphia chromosome. Among these are the association of deletions of genes on derivative (der) 9 chromosome with adverse outcomes in CML patients. However, the functional role of genes near the breakpoint on der (9) in CML prognosis and progression remains largely unexplored. Copy number variation and mRNA expression were evaluated for five genes located near the breakpoint on der (9). Our data showed a significant association between microdeletions of the FUBP3 gene and its reduced expression with poor prognostic markers and adverse response outcomes in CML patients. Further investigation using K562 cells showed that the decrease in FUBP3 protein was associated with an increase in proliferation and survival due to activation of the MAPK–ERK pathway. We have established a novel direct interaction of FUBP3 protein and PRC2 complex in the regulation of ERK signalling via PAK1. Our findings demonstrate the role of the FUBP3 gene located on der (9) in poor response and progression in CML with the identification of additional druggable targets such as PAK1 in improving response outcomes in CML patients.

## 1 INTRODUCTION

Chronic myeloid leukaemia (CML) is a clonal myeloproliferative disorder characterized by a reciprocal translocation t(9;22)(q34;q11) between ABL1 and BCR gene on chromosome 9 and 22, respectively.^1^ The translocation results in the concomitant formation of the Philadelphia (Ph) 22q‐ and derivative (der) 9q+ chromosome.^1^ The BCR–ABL1 fusion protein on the Ph chromosome; a constitutively active tyrosine kinase, has been reported to be the underlying factor in the pathogenesis of CML,^2^ which leads to perturbation of key signalling pathways resulting in increased proliferation and survival.^2^, ^3^ In the last two decades, the development of various tyrosine kinase inhibitors has revolutionized the treatment of CML.^4^ However, the biggest limitation in the management of CML has been the development of resistance and differential response to TKI. This can be attributed to mechanisms directly affecting or independent of BCR–ABL1 protein.^5^, ^6^, ^7^ The transformation of the disease to advanced stages leads to the clonal evolution of leukaemic stem cells with the acquisition of additional chromosomal abnormalities such as extra Ph+ chromosome, trisomy 8 and isochromosome 17q.^8^, ^9^ The deletions of genes near the ABL1–BCR breakpoint on der (9) have also been implicated in poor response outcomes in CML patients.^10^, ^11^, ^12^ The patients harbouring these deletions had shorter median Overall Survival (OS) and Event Free Survival (EFS).^10^, ^11^, ^12^ The deletions were reported to occur early and possibly at the time of the Ph chromosome translocation.^13^ However, certain groups observed no significance between der(9) deletions and poor prognosis^14^, ^15^ in CML patients. In the Indian population, the deletions on der(9) were found to be associated with an unfavourable TKI response.^16^, ^17^

The role of the Philadelphia chromosome in the development and management of CML has been well explored. Owing to the considerable heterogeneity in prognosis and progression of CML given its homogeneous background of Ph chromosome, it is imperative to understand the role of der (9) in the pathophysiology of CML. The aforementioned studies were limited in exploring the molecular and biological mechanism of genes on der (9) leading to therapy failure. This study was therefore designed to understand the functional contribution of the genes near the ABL1–BCR breakpoint on der (9) in the progression and prognosis of CML.

## 2 MATERIALS AND METHODS

### 2.1 Collection of samples

In total, 84 CML patients and 16 healthy control samples were recruited for the study as per the approval by the IEC at the St. John's Medical College and Hospital, Bengaluru (IERB no. 252/2016). Male (*n* = 48) and female (*n* = 36) patients between 18–65 years (median age 40 years) with a confirmed diagnosis of BCR–ABL positive CML were included in the study. Peripheral blood and/or bone marrow (BM) samples were collected at the time of diagnosis and/or during the loss of TKI response in patients upon follow‐up. Out of 84 patients, 72 were in the chronic phase, two were in the accelerated phase, and nine in blast crisis (Table 1). According to the current ELN guidelines,^18^ fifty‐three patients showed an optimal response, 14 patients showed a warning response, and 17 patients showed the failure of response (Table 1). Sixty‐six patients were on a standard dose of imatinib 400 mg/day, nine patients were on imatinib 600 mg/day, eight patients were on dasatinib 100 mg/day, and three patients were on nilotinib 300 mg twice daily (Table 1). Sokal, Hasford and EUTOS scores were calculated for each patient and used to estimate survival and response risk at baseline^19^ (Table 1). The baseline characteristics of the CML patients are listed in Table 1.

**TABLE 1:** Baseline characteristics of CML patients (*N* = 84)

Gender	
Male	48
Female	36
Male:Female	1.33:1
Age (Years)	
Mean	41
Median	40
Range	(18–65)
Platelets (×10^9^/L)	
Mean	360.5
25% Percentile	203.8
Median	274.5
75% Percentile	385
Range	(53–1397)
Haemoglobin (g/dl)	
Mean	10.02
25% Percentile	8.3
Median	10.25
75% Percentile	11.8
Range	(5.2–15.1)
Total counts (×10^9^/L)	
Mean	156.2
25% Percentile	46.5
Median	127
75% Percentile	223
Range	(3.83–589.38)
Spleen (cm)	
Mean	16.74
25% Percentile	13
Median	15
75% Percentile	20
Range	(10–35)
Basophils (%)	
Mean	3.821
25% Percentile	1.25
Median	3
75% Percentile	5
Range	(0–23)
Eosinophils (%)	
Mean	4.893
25% Percentile	2
Median	4
75% Percentile	7
Range	(0–16)
Myeloblasts (%)	
Mean	3.976
25% Percentile	0.25
Median	2
75% Percentile	4
Range	(0–59)
Stage of disease	
Chronic Phase	72
Accelerated Phase	2
Blast Crisis	9
Response	
Optimal	53
Warning	14
Failure	17
TKI treatment	
Imatinib 400 mg	66
Imatinib 600 mg	9
Dasatinib 100 mg	8
Nilotinib 300 mg	3
Bone marrow fibrosis	
No	32
Mild	28
Marked	24
Sokal	
Low	49
Intermediate	27
High	8
Hasford	
Low	59
Intermediate	25
EUTOS score	
Low	73
High	11

### 2.2 DNA and RNA isolation and cDNA preparation

DNA was isolated from peripheral blood and/or BM samples by salting out. RNA was isolated using TriZol™ (Invitrogen, Thermo Scientific) as per the manufacturer's instructions. 1 μg RNA was converted to cDNA using an M‐MLV reverse transcriptase kit (Invitrogen, Thermo Scientific).

### 2.3 Real time quantitative PCR (RT‐PCR)

#### 2.3.1 SYBR green assay

The reaction included 300 nM forward and reverse primer, 20 ng DNA (Copy Number Variation [CNV] assay)/1 μl (1:10 diluted) cDNA (gene expression assay), 2X TB Green Premix Ex Taq II (Takara Bio; RR820B). Each sample was run in triplicates. The run was performed on ViiA‐7 or 7500‐Fast RT‐PCR (Applied Biosystems, Thermo Scientific). GAPDH was used as the housekeeping gene. The sequences for primers used are listed in Table S1. Standards for quantification were prepared using PCR and purified using a PCR clean‐up kit (Promega). Standards ranging from 10^6^ copies/μl to 10 copies/μl for target genes were included with every reaction setup. The target quantity was determined by interpolating from the standard curve. The test and calibrator samples were normalized by their respective endogenous control. Fold change in the target gene was calculated by dividing the normalized target (test sample) by the normalized target (calibrator sample) and/or by ΔΔC_T_ method. For the CNV assay, the copy number value of 1 indicated normal values. The values in the range of 0.5 and below were considered as deletion, and values 1.5, and above were considered as duplication.

#### 2.3.2 Taq‐Man assay

Taq‐CNV assay for FUBP3 gene (reporter: FAM dye, quencher: MGB–NFQ) and reference gene RNaseP (reporter: VIC, quencher: TAMRA) (Applied Biosystems, Thermo Scientific) was performed as per the manufacturer's instructions on 7500‐Fast RT‐PCR (Applied Biosystems, Thermo Scientific). Copy numbers were calculated using CopyCaller™ software (Applied Biosystems, Thermo Scientific). The copy number value of 2 indicated normal values. The values close to 1 were considered as deletions.

### 2.4 Cell lines and reagents

Chronic myeloid leukaemia cell line K‐562 was cultured in RPMI‐1640 media (Invitrogen, Thermo Scientific) with 10% FBS (HiMedia) at 37°C in 5% CO_2_. Imatinib mesylate (Sigma–Aldrich; SML1027), dasatinib (Sigma–Aldrich, SML2589), and bosutinib (Bonitar 100 mg) were used as inhibitors for BCR‐ABL1 protein. PD184352 (Sigma–Aldrich; PZ0181), A‐395 (Adooq Biosciences; A12999), and IPA‐3 (Sigma–Aldrich; I2285) were used as inhibitors for MEK/ERK kinase, PRC2 complex, and PAK1 kinase respectively. Antibodies used were FUBP3 (sc‐398,466; E8), GAPDH (CST‐14C10), Histone H3 (CST‐D1H2), beta‐tubulin (Sigma–Aldrich N6786), p44/42 MAPK (ERK1/2) (CST‐137F5), phospho‐p44/42 MAPK (ERK1/2) (CST‐D13.14.4E), p38 MAPK (CST‐D13E1), phospho‐p38 MAPK (CST‐D3F9), SAPK/JNK (CST‐9252), phospho‐SAPK/JNK (CST‐81E11), SUZ12 (CST‐D39F6), EZH2 (CST‐D2C9), EED (CST‐E4L6E), RBBP4/7 (RBAP48/RBAP46) (CST‐4633), Mouse IgG (CST‐G3A1), Mouse HRP (Sigma–Aldrich A3682) and Rabbit HRP (Sigma–Aldrich AQ132P).

### 2.5 Immunoblotting

The cells were lysed using RIPA buffer (20 mM Tris–HCl (pH ‐7.5), 150 mM NaCl, 1% Triton X‐100, 0.1% (w/v) SDS, 2 mM EDTA, 10 mM NaF, 1 mM Na_3_VO_4_, 1 mM PMSF, 1X MPI cocktail). The proteins were resolved using SDS–PAGE and detected using an ECL reagent (Pierce, Thermo Scientific).

### 2.6 Co‐immunoprecipitation

The protein G Dynabeads (Invitrogen, Thermo Scientific) were used as per the manufacturer's instructions and equilibrated by mixing with lysis buffer (20 mM Tris–HCl [pH 7.5], 150 mM NaCl, 1% Triton X‐100, 1% NP‐40, 2 mM EDTA, 10 mM NaF, 1 mM Na_3_VO_4_, 1 mM PMSF, 1X MPI cocktail). Equilibrated Dynabeads were mixed with 1 μg antibody of protein of interest and 1 μg of isotype antibody for 30 minutes at RT. The antibody‐conjugated Dynabeads were mixed with pre‐cleared lysate and incubated overnight at 4°C followed by protein elution using 1X lamelli buffer at 95°C for 5 minutes.

### 2.7 Transfection

50 pmol FUBP3 and scrambled siRNA (Invitrogen, Thermo Scientific), were transfected in K562 cells using HiPerFect (Qiagen) reagent as per manufacturer's instructions. After 48 hours, cells were retrieved for further assays.

### 2.8 Colony forming unit (CFU) assay

1% methylcellulose (Sigma–Aldrich) was prepared in RPMI and 15% FCS as per the manufacturer's instructions. 2000 cells were seeded per 35 mm dish in 1.5 ml of methylcellulose media. The dishes were incubated at 37°C under 5% CO_2_. The colonies were counted after 14 days.

### 2.9 Cell viability assay

1 × 10^4^ cells were seeded per well in a 96‐well plate with or without inhibitors. After 48 h 5 μl WST‐1 reagent (Roche) was added per well, followed by a 30‐min incubation. The absorbance was measured at 450 nm with background reduction at 655 nm using ELISA microplate reader (Bio‐Rad Laboratories).

### 2.10 Transcriptomic analysis

RNA from FUBP3 and scrambled siRNA‐treated cells (*n* = 3) were subjected to Illumina paired‐end sequencing (150 × 2). Sequenced reads were aligned to *Homo sapiens* DRCh38 build genome from Ensembl.^20^ HISAT2^21^ and HTSeq^22^ were used to align transcript sequences and calculate the change in expression. DGE analysis was performed using DESeq.^23^ Differentially expressed genes were calculated using ±1.5‐fold change and *p* ≤ 0.05. DAVID 6.8 was used for gene ontology (GO) analysis.^24^ STRING database v11^25^ and ClustVis^26^ was used for creating protein network and heatmaps, respectively.

### 2.11 Mass spectrometry

FUBP3 protein was immunoprecipitated from K562 nuclear fraction^27^ using protein G Dynabeads (Invitrogen, Thermo Scientific). The beads were subjected to mass spectrometry. The fractions were analysed on an Orbitrap Fusion Tribrid mass spectrometer (Thermo Scientific). The scan range was set at 400–1600 (m/z), and analyser was operated at a mass resolution of 120,000 at the MS level. The data acquired was searched against the NCBI Human RefSeq81 protein database. The searches were performed using SEQUEST and MASCOT through Proteome Discoverer™ (version 2.1) software suite (Thermo Scientific). The protein network was constructed using Cytoscape v3.9.0.^28^

### 2.12 Chromatin immunoprecipitation

SimpleChIP kit (CST; 9003) was used for ChIP assay as per the manufacturer's protocol. 1 μg FUBP3 antibody was used, and histone and IgG controls were set up. The eluted DNA was sent for 150 paired‐end sequencing in duplicates along with input DNA. The ChIP‐seq libraries were prepared with NEXTflex ChIP‐seq library prep kit (Bio Scientific). The raw data was processed using Bowtie^29^ and aligned with the reference human genome. The peak calling and motif identification was done by HOMER.^30^

### 2.13 cBioPortal analysis

The effect of altered expression of FUBP3 and PAK1 on the OS in clinical samples and the correlation between FUBP3 and PAK1 expression was analysed by cBioPortal database^31^ using acute myeloid leukaemia study.^32^

### 2.14 Statistical analysis

Pearson's and/or Spearman's rho correlation was performed to measure the association between two variables. For parametric data, unpaired sample *t*‐test and one‐way analysis of variance (anova) were performed to analyse the mean difference between two or more groups. Following a significant anova, planned contrasts were also performed to compare means between specific groups. A set of weights were developed to eliminate the means of any group that were not involved in the comparison by giving them a zero weight, and the group means to be compared were specified by giving them opposite values. For nonparametric data, the Mann–Whitney *U* test and the Kruskal–Wallis test were performed to analyse median difference between two or more groups. Tukey's and Dunn's tests were used as multiple comparison tests. *p* < 0.05 was considered statistically significant. All the tests were performed using GraphPad Prism 9 and/or SPSS software.

## 3 RESULTS

### 3.1 Microdeletions of FUBP3 gene are associated with poor TKI response in CML patients

**FIGURE 1:**
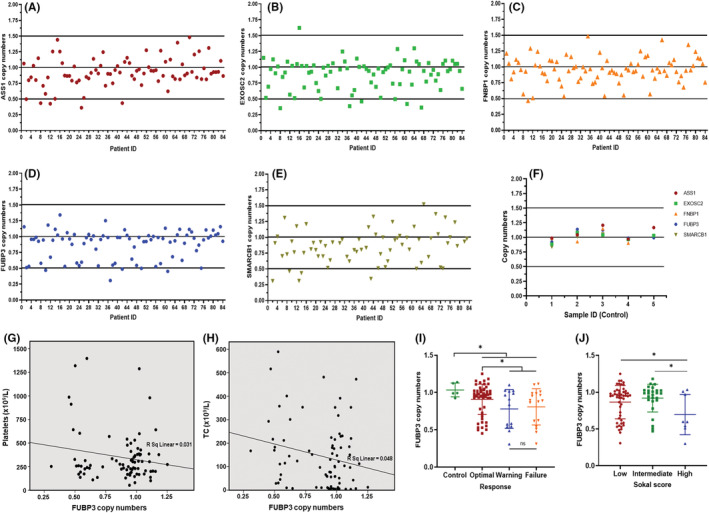
Copy numbers of target genes on der (9) in CML and control samples and the significant association of copy numbers of FUBP3 gene with clinical parameters in CML patients. Scatter plot representing the DNA copy numbers of (A) ASS1 (B) EXOSC2 (C) FNBP1 (D) FUBP3 (E) SMARCB1 gene in individual patient sample detected by RT‐PCR. The copy number value of 1 indicated normal values. The values in the range of 0.5 and below were considered as deletion and values 1.5 and above were considered as duplication (F) Copy numbers of ASS1, EXOSC2, FNBP1, FUBP3 and SMARCB1 genes in five control samples detected by RT‐PCR (G) Negative correlation between platelet counts (×10^9^/L) and copy numbers of FUBP3 gene in CML patients (Pearson's *r* = −0.254, *p* ≤ 0.05) (H) Negative correlation between total counts (TC) (×10^9^/L) and copy numbers of FUBP3 gene in CML patients (Pearson's *r* = −0.244, *p* ≤ 0.05) (I) Copy numbers of FUBP3 gene in control group and CML patients with optimal, warning and failure TKI response. Each data point represents DNA copy number. Horizontal lines represent mean ± SD. *: *p* ≤ 0.05, ns: nonsignificant (J) Copy numbers of FUBP3 gene in CML patients with low, intermediate, and high Sokal score. Each data point represents DNA copy number. Horizontal lines represent mean ± SD *: *p* ≤ 0.05

CNV analysis was performed for five genes; Arginosuccinate synthase 1 (ASS1), Exosome component 2 (EXOSC2), Far upstream binding protein 3 (FUBP3), Formin binding protein 1 (FNBP1), and SWI/SNF Related Matrix Associated Actin Dependent Regulator of Chromatin (SMARCB1) (Table S2) in CML and control samples using RT–PCR. ASS1 gene has been implicated in the migration and invasion of endometrial tumors^33^ and poor outcomes in gastric cancer.^34^ The mutations of the EXOSC2 gene are shown to be associated with SHRF disorder.^35^ FNBP1 and its translocation with MLL has been reported in paediatric AML response.^36^ FUBP3 belongs to a family of far upstream binding proteins reported to interact with DNA or RNA targets and regulate transcription, splicing and translation.^37^ SMARCB1 is a tumour suppressor, and its mutations have been associated with malignant transformation in rhabdoid tumors.^38^ Out of eighty–four CML patients, the microdeletions of ASS1, EXOSC2, FNBP1 and FUBP3 genes were detected in seven (8.3%), twelve (14.2%), seven (8.3%), and nineteen (22%) patients, respectively (Figure 1A–D). The microdeletions of the SMARCB1 gene were detected in twelve out of sixty–eight (17.6%) patients (Figure 1E). Figure 1F shows the copy numbers of selected genes in five control samples.

Correlation between clinical parameters and copy numbers of ASS1, EXOSC2, FNBP1, FUBP3 and SMARCB1 genes (Table S3) revealed a significant association of FNBP1 copy numbers with basophils (*r* = 0.295, *p* = 0.007) and platelet counts (*r* = −0.266, *p* = 0.015). There was also a significant negative correlation between copy numbers of FUBP3 gene with high platelet counts (*r* = −0.254, *p* = 0.020) and high total counts (TC) (*r* = −0.244, *p* = 0.029) (Figure 1G,H; Table S3).

anova analysis of copy numbers of five genes and TKI response suggested that only the copy numbers of the FUBP3 gene showed a significant association with TKI response (*p* = 0.023) (Figure 1I). Planned contrast analysis showed a significant difference in FUBP3 copy numbers (*p* = 0.003) between patients in the control group and those in optimal, warning and failure groups combined (Figure 1I). Also, patients in the warning and failure response group combined showed a significant reduction in FUBP3 copy numbers as compared to those in optimal group (*p* = 0.034) (Figure 1I). anova analysis also revealed a significant difference in FUBP3 copy numbers in patients with different Sokal scores (*p* = 0.022). The planned contrast studies showed a significant association of low FUBP3 copy numbers in patients with high Sokal scores as compared to patients with intermediate (*p* = 0.006) and low scores (*p* = 0.020) (Figure 1J). A high Sokal score, indicative of poor prognosis in CML patients, plays an important role in assigning risk levels to patients at baseline.^39^ There was no significant difference in FUBP3 copy numbers in patients with low and high Hasford (*p* = 0.414) and EUTOS (*p* = 0.787) score (Figure S1).

### 3.2 FUBP3 expression is downregulated in CML patients and is associated with poor clinical response outcomes

**FIGURE 2:**
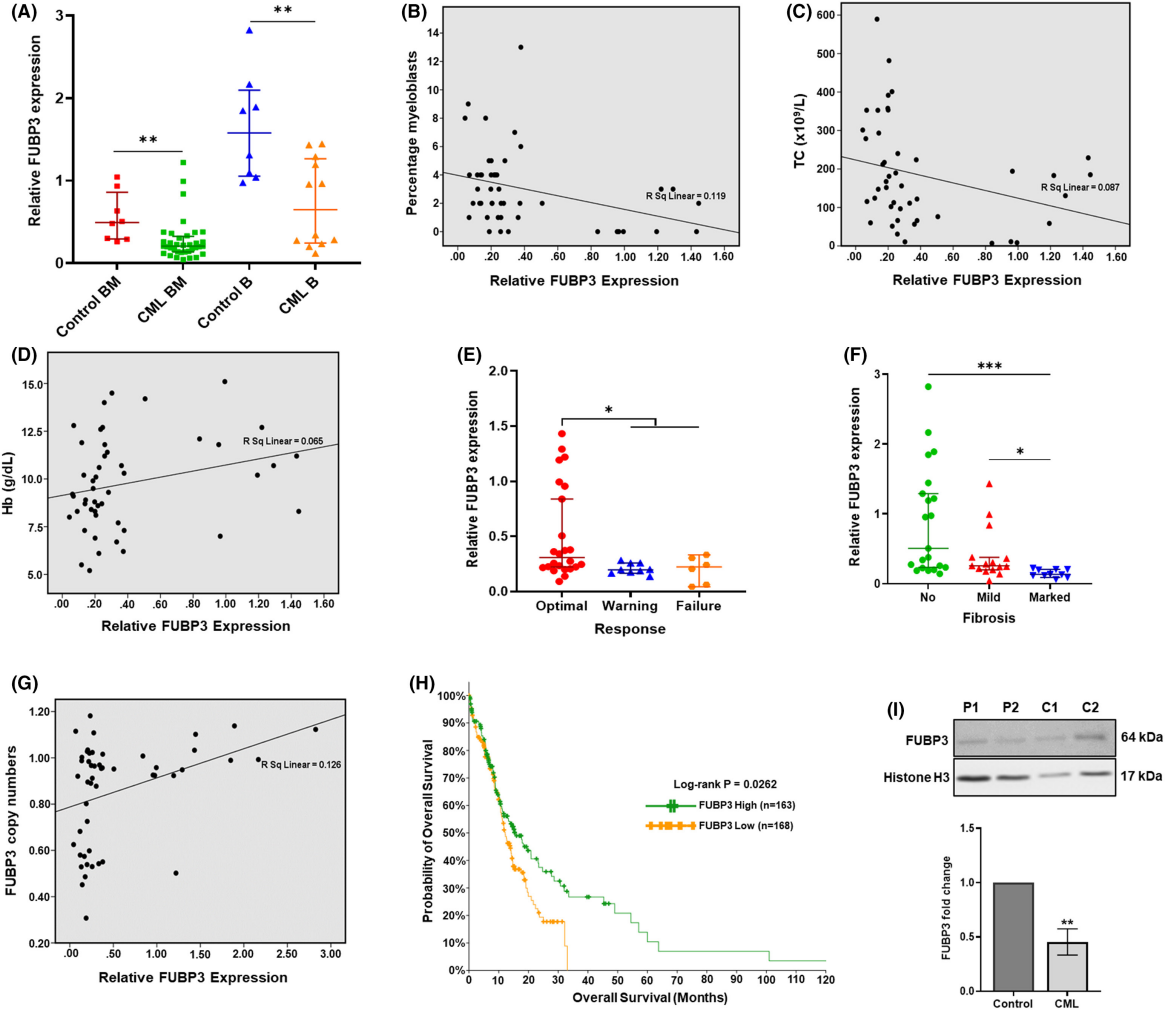
FUBP3 expression is downregulated in CML patients and is associated with poor clinical response outcomes (A) The expression of FUBP3 gene is significantly reduced in CML patient samples as compared to control samples as determined by RT‐PCR. Each data point represents normalized mRNA expression. Horizontal lines represent median ± interquartile range. BM, bone marrow; B, blood, ** *p* ≤ 0.01 (B) Negative correlation between the percentage of myeloblasts and relative mRNA expression of FUBP3 gene in CML patients (Spearman's *r* = −0.345; *p* ≤ 0.05) (C) Negative correlation between total counts (TC) (x10^9^/L) and relative mRNA expression of FUBP3 gene in CML patients (Spearman's *r* = −0.439, *p* ≤ 0.01) (D) Positive correlation between haemoglobin levels (Hb) (g/dl) and relative mRNA expression of FUBP3 gene in CML patients (Spearman's *r* = 0.294, *p* ≤ 0.05) (E) Relative mRNA expression of FUBP3 gene in CML patients with optimal, warning and failure TKI response. Each data point represents normalized mRNA expression. Horizontal lines represent median ± 95% class interval. * *p* ≤ 0.05 (F) Relative mRNA expression of FUBP3 gene in CML patients with no, mild and marked bone marrow fibrosis. Each data point represents normalized mRNA expression. Horizontal lines represent median ± 95% class interval. *** *p* ≤ 0.001, * *p* ≤ 0.05 (G) Positive correlation between copy numbers and mRNA expression of FUBP3 gene in CML patients (Spearman's *r* = 0.369, *p* ≤ 0.01) (H) Kaplan–Meier survival curve indicating the decrease in median overall survival of patients with low (yellow) FUBP3 expression (12.17 months) as compared to those with high (green) FUBP3 expression (15.46 months) (I) Representative western blot depicting a decrease in FUBP3 protein levels in CML samples as compared to control. Histone H3 was used as a loading control. L–R: P1:CML sample 1, P2:CML sample 2, C1:control sample 1, and C2:control sample 2. Bar graph representing the fold change decrease in FUBP3 protein level in CML samples compared to control samples. Data represented as mean ± SD (*n* = 3). **: *p* ≤ 0.01

The mRNA expression of ASS1, EXOSC2, FNBP1, SMARCB1 and FUBP3 genes was evaluated in forty‐eight CML and sixteen control samples. There was a difference in the median expression of target genes in blood and BM samples, thus, the samples were segregated for statistical analysis. The Mann–Whitney U test showed a significant decrease in FUBP3 expression in CML samples compared to control samples in BM (*p* = 0.0012) as well as blood‐derived samples (*p* = 0.0073) (Figure 2A). The difference in EXOSC2 expression was also significant in both BM (*p* = 0.0066) and blood (*p* = 0.0011) samples (Figure S2A). The difference in ASS1 (*p* = 0.0375) and FNBP1 (*p* = 0.0016) expression was significant only in BM‐derived samples and not in blood samples (*p* = 0.0574) (*p* = 0.0979), respectively (Figure S2B,C). SMARCB1 expression was nonsignificant in both BM (*p* = 0.0604) and blood samples (*p* = 0.0825) (Figure S2D).

Since there was a significant difference in FUBP3 and EXOSC2 expression in control and CML samples in both blood and BM samples, we chose these genes for further analysis. The decrease in FUBP3 expression was significantly associated with a higher percentage of myeloblasts (*r* = −0.345, *p* = 0.016), high TC (*r* = −0.439, *p* = 0.002) and low Hb levels in CML patients (*r* = 0.294, *p* = 0.043) (Figure 2B–D; Table S4). EXOSC2 expression also showed a significant negative correlation with TC (*r* = −0.447, *p* = 0.001) and percentage of myeloblasts (*r* = −0.304, *p* = 0.035) in patient samples (Figure S3A,B), but there was no significant difference in its expression between different TKI response groups (*p* = 0.3143) (Figure S3C).

The decrease in FUBP3 expression was significantly associated with poor TKI response in CML patients (*p* = 0.0224) (Figure 2E). The difference in FUBP3 expression was also significantly associated with BM fibrosis (*p* = 0.0002). Patients with marked fibrosis had a significant reduction in FUBP3 expression as compared to patients with no (*p* = 0.0001) and mild fibrosis (*p* = 0.0319) (Figure 2F). Furthermore, a significant positive association was observed between copy numbers and expression of the FUBP3 gene (*r* = 0.369, *p* = 0.008) (Figure 2G; Table S5). Since the patients recruited in our study are yet to complete 5 years, we used cBioPortal to understand if FUBP3 expression has any effect on OS in leukaemias, including CML. There is no large CML dataset available on the portal, however, analysis of acute leukaemic samples revealed a significant decrease in the median OS of patients with low FUBP3 expression (12.17 months) compared to those with high FUBP3 expression (15.46 months) (*p* = 0.0262) (Figure 2H). Upon performing western blot analysis, we also observed a significant 0.45‐fold reduction in FUBP3 protein levels in CML samples (*p*≤  0.01) (Figure 2I). The microdeletions of FUBP3 gene were validated using TaqMan assay and were compared with SYBR Green RT–PCR data (Figure S4A). However, for four patients with deletions, the assay could not be performed due to the unavailability of the sample.

Among the three proteins of the FUBP family, the role of FUBP1 has been well characterized in haematopoiesis, embryonic development and haematological malignancies. FUBP1^−/−^ knockout mice are embryonic lethal.^40^ FUBP1 was found to be overexpressed in murine leukaemia models, with its knockdown resulting in increased survival and apoptosis.^41^ The role of FUBP3 has not been characterized in leukaemia, and to comprehend the same, FUBP3‐siRNA mediated knockdown experiments were performed in K562 cells to mimic the conditions in CML samples. There was no off‐target effect of FUBP3 siRNA on the expression of other genes of the family, i.e., FUBP1 and FUBP2 (Figure S4B).

**FIGURE 3:**
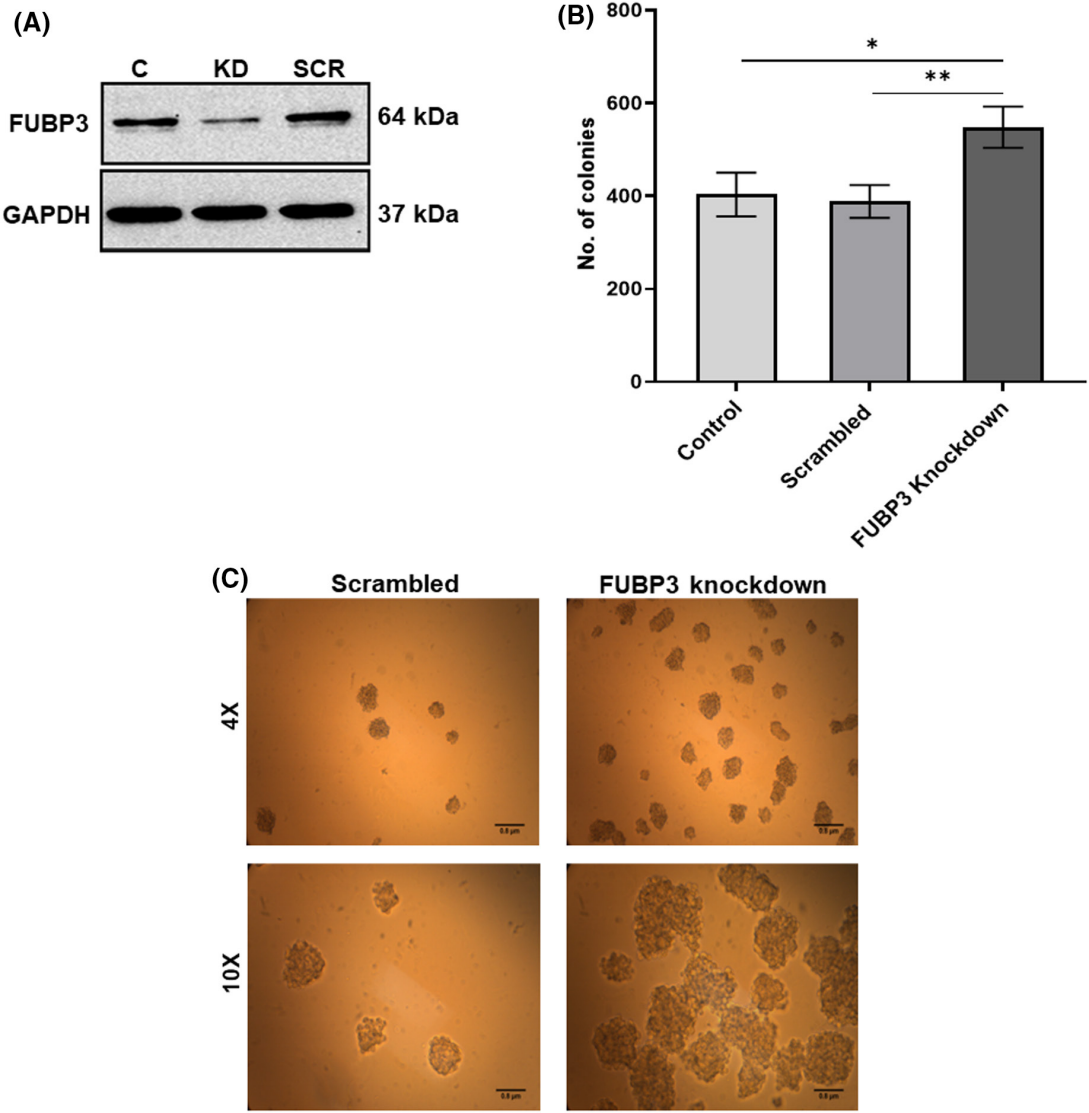
FUBP3 knockdown results in a gain of phenotype in K562 cells (A) Representative image of western blot depicting the reduction in the levels of FUBP3 protein post siRNA treatment in K562 cells. GAPDH was used as a loading control. L‐R: C: no siRNA, KD: FUBP3 siRNA, SCR: scrambled siRNA (B) The increased number of colonies formed by FUBP3 knockdown cells as compared to scrambled and no siRNA control cells in CFU assay. Data represented as mean ± SD (*n* = 3). **: *p* ≤ 0.01, *: *p* ≤ 0.05, ns: nonsignificant (C) Representative images showing an increase in size and numbers of colonies formed by FUBP3 knockdown cells compared to scrambled from CFU assay at 4X and 10X magnification; Scale bar = 0.8 μm.

Clonogenic CFU assay was performed to understand the functional effect of FUBP3 knockdown on the phenotype of K562 cells wherein FUBP3 knockdown cells formed a significantly higher number of colonies compared to scrambled (*p* ≤ 0.01) and no siRNA control (*p* ≤ 0.05) (Figure 3A,B). The colonies generated by FUBP3 knockdown cells indicated a gain in phenotype displayed by larger size and increased proliferation as compared to colonies formed by scrambled cells (Figure 3C).

### 3.3 Global transcriptional regulation by FUBP3 gene

**FIGURE 4:**
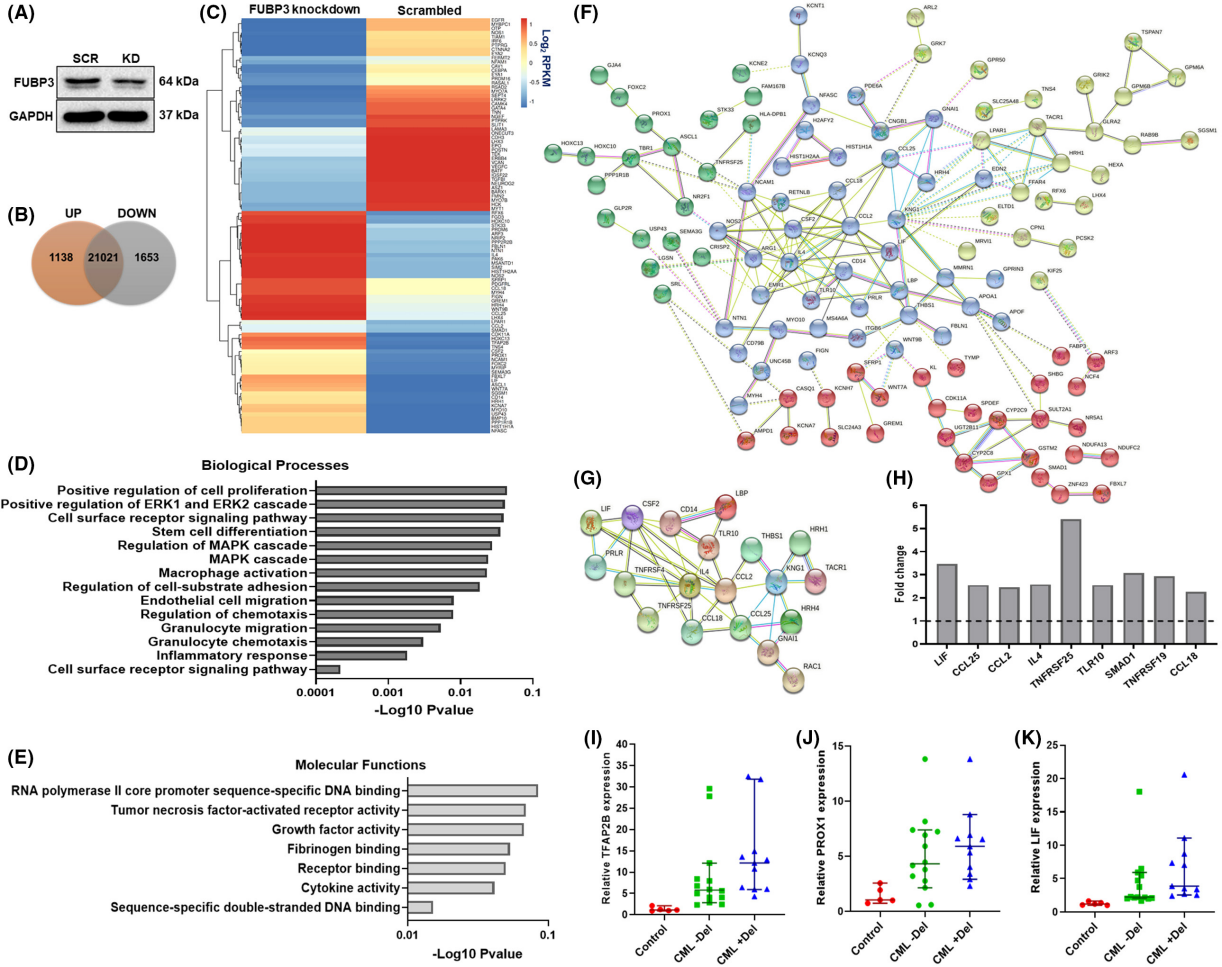
Global transcriptomic networks regulated by FUBP3 gene (A) Representative image of a western blot showing the reduction in the level of FUBP3 protein in K562 cells subjected to transcriptomic sequencing upon siRNA knockdown. GAPDH protein was used as a loading control. L‐R: SCR: scrambled siRNA and KD: FUBP3 siRNA (B) Venn diagram representing the numbers of upregulated (orange), downregulated (grey) and neutrally expressed genes in the FUBP3 knockdown cells compared to scrambled (C) Heatmap representing the hierarchical clustering of differentially expressed genes in FUBP3 knockdown (KD) and scrambled cells sorted using log2 RPKM values (D) GO analysis of biological processes for upregulated genes enriched in FUBP3 knockdown cells with *p* < 0.05 and their corresponding ‐Log10 *p*‐value scores (E) GO analysis of molecular functions for upregulated genes enriched in FUBP3 knockdown cells with *p* < 0.05 and their corresponding ‐Log10 *p*‐value scores (F) A protein network of 182 upregulated genes enriched in FUBP3 knockdown cells generated using STRING dB v11. Four clusters were identified using the k‐means approach and are denoted by a separate colour. The network showed enrichment of processes involved in the regulation of cell proliferation, MAPK cascade, migration and inflammatory response. Each node represents a protein (G), A protein network of 19 upregulated genes involved in fibrosis enriched in FUBP3 knockdown cells generated using STRING dB v11 at minimum confidence 0.4. Each node represents a protein (H) The fold difference in the expression of key upregulated genes involved in fibrosis in FUBP3 knockdown cells over scrambled control from the transcriptomic data analysis (I) Relative mRNA expression of TFAP2B gene in control samples and patients without (CML Del) and with (CML + Del) deletion of FUBP3 gene detected by RT‐PCR. Each data point represents normalized mRNA expression. Horizontal lines represent median ± 95% class interval (J) Relative mRNA expression of PROX1 gene in control samples and patients without (CML ‐Del) and with (CML + Del) deletion of FUBP3 gene detected by RT‐PCR. Each data point represents normalized mRNA expression. Horizontal lines represent median ± 95% class interval (K) Relative mRNA expression of the LIF gene in control samples and patients without (CML ‐Del) and with (CML + Del) deletion of FUBP3 gene detected by RT‐PCR. Each data point represents normalized mRNA expression. Horizontal lines represent median ± 95% class interval.

Since FUBP3 protein reduction in K562 cells showed a strong gain of phenotype, it was imperative to understand the molecular pathways regulated by FUBP3 in this context. We performed transcriptomic analysis of K562 cells post FUBP3 knockdown to identify the gene regulatory pathways modulated by the FUBP3 gene (Figure 4A). The differential expression of genes between scrambled and FUBP3 knockdown cells is shown in Figure 4B,C. The GO analysis of enriched upregulated genes revealed modification of diverse processes involved in the regulation of cell proliferation, MAPK cascade, adhesion, migration, chemotaxis, inflammatory response, cytokine activity and DNA binding (Figure 4D,E). Based on DAVID GO analysis, the upregulated genes were further filtered down to 182 key genes, and the protein interaction between them was analysed using the STRING database (Figure 4F). The downregulated genes were further enriched for 124 genes, and their interaction revealed distinct clusters involved in the positive regulation of MAPK signalling, mRNA splicing, cell proliferation and cell adhesion (Figure S5A). The fold change decrease in the expression of key genes in knockdown cells from this protein cluster is depicted in Figure S5B. GO analysis of FUBP3 knockdown cells also showed an enrichment and upregulation of genes involved in fibrosis (Figure 4G,H). The expression of some of the key upregulated and downregulated genes from the transcriptomic analysis were validated in both the K562 cell line as well as patient samples and showed comparable results (Figure 4I–K; Figure S6,S7).

### 3.4 FUBP3 regulates the MAPK–ERK pathway

**FIGURE 5:**
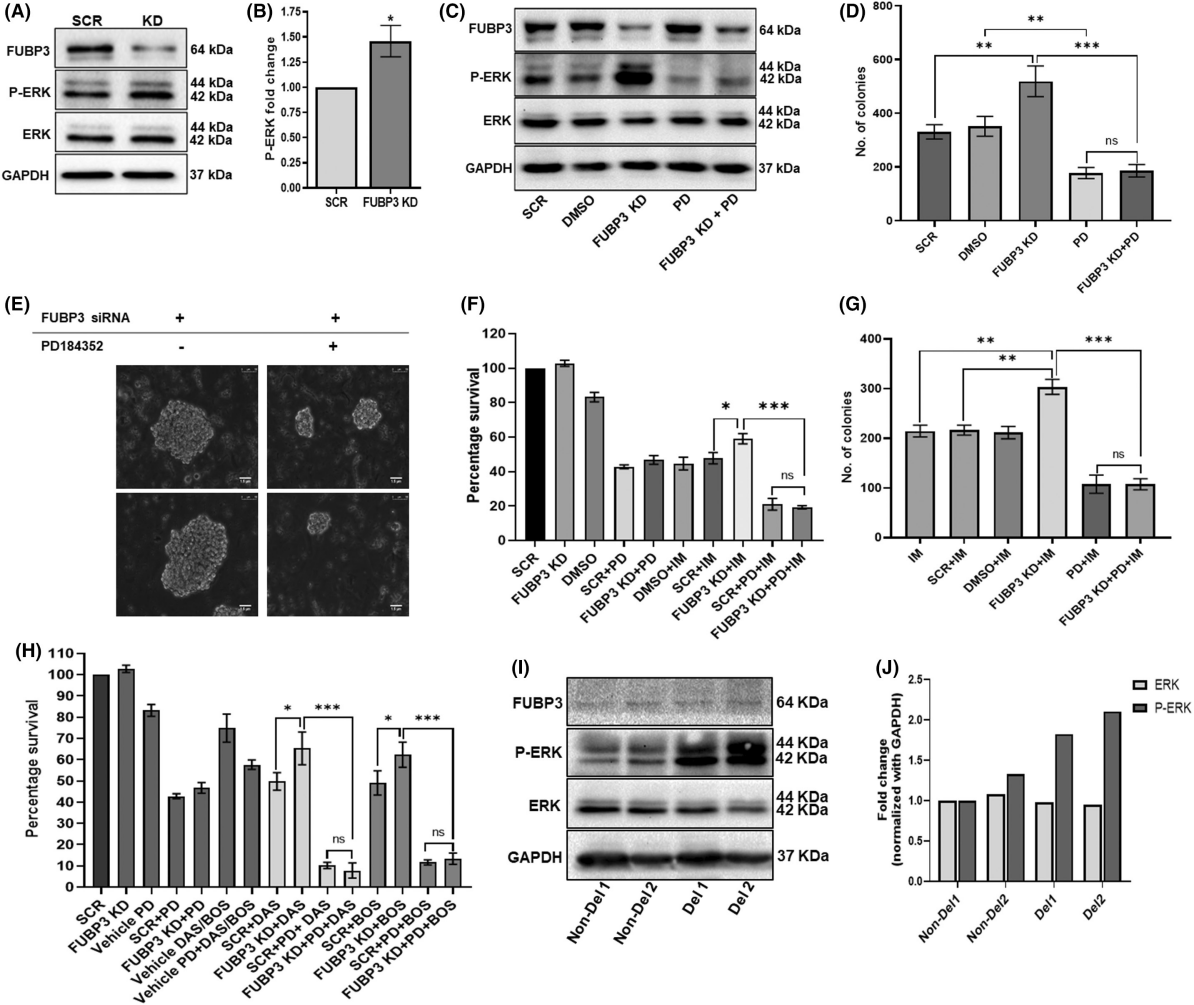
Downstream regulation of MAPK–ERK pathway by FUBP3 protein (A) Representative image of western blot indicating increase in levels of phospho‐ERK1/2 (p‐ERK1/2) protein post FUBP3 knockdown. GAPDH was used as a loading control. L‐R: SCR: scrambled siRNA and KD: FUBP3 siRNA (B) FUBP3 knockdown results in a significant increase in the level of phospho‐ERK1/2 protein compared to the scrambled control. Data represented as mean ± SD (*n* = 3) *p ≤ 0.01. SCR: scrambled siRNA, FUBP3 KD: FUBP3 siRNA (C) Representative image of western blot indicating a decrease in the level of p‐ERK1/2 protein in FUBP3 knockdown cells after treatment with ERK inhibitor PD184352. L‐R: SCR: scrambled siRNA, DMSO (v/v): vehicle for PD184352, FUBP3 KD: FUBP3 siRNA, PD: 10 μM PD184352, FUBP3 KD + PD: FUBP3 siRNA plus 10 μM PD184352 (D) The treatment of FUBP3 knockdown cells with ERK inhibitor PD184352 abrogates the gain in phenotype in these cells denoted by the reduction in the number of colonies formed by these cells in CFU assay. L‐R: SCR: scrambled siRNA, DMSO (v/v) vehicle for PD184352, FUBP3 KD: FUBP3 siRNA, PD: 10 μM PD184352, FUBP3 KD + PD: FUBP3 siRNA plus 10 μM PD184352. Data are expressed as mean ± SD (*n* = 3). *** *p* ≤ 0.001, **: *p* ≤ 0.01, ns: nonsignificant (E) Representative images showing a reversal in the gain of phenotype of colonies formed by FUBP3 knockdown cells upon ERK inhibition by PD184352 in CFU assay under 20X magnification. Scale‐1.5 μm (F) Cell viability represented as percentage survival of K562 cells assessed by WST‐1 assay. The gain in survival of FUBP3 knockdown cells post 0.5 μM imatinib (IM) treatment alone was abrogated by their combined treatment with 10 μM PD184352 (PD). SCR: scrambled siRNA, FUBP3 KD: FUBP3 siRNA, DMSO (v/v) vehicle for PD184352. Data are expressed as mean ± SD (*n* = 3). ***p ≤ 0.001, *p ≤ 0.05, ns: nonsignificant (G) Number of colonies formed by K562 cells under different conditions of treatment with 0.2 μM imatinib (IM) and PD184352 in CFU assay. L‐R: IM: no siRNA control, SCR: scrambled siRNA, DMSO (v/v) vehicle for PD184352, FUBP3 KD: FUBP3 siRNA, PD: 10 μM PD184352, FUBP3 KD + PD: FUBP3 siRNA plus 10 μM PD184352. Data are expressed as mean ± SD (*n* = 3). ***: *p* ≤ 0.001, **p ≤ 0.01, ns: nonsignificant (H) Cell viability represented as percentage survival of K562 cells assessed by WST‐1 assay. The increased survival of FUBP3 knockdown cells post TKI 0.6 nM dasatinib (DAS), and 40 nM bosutinib (BOS) treatment alone was drastically subsided upon combinatorial treatment of these cells with ERK inhibitor 10 μM PD184352 (PD). 2 μl, 3 μl, and 5 μl DMSO (v/v) was used as a vehicle for PD, DAS/BOS, and PD + DAS/BOS respectively. SCR: scrambled siRNA, FUBP3 KD: FUBP3 siRNA. Data represented as mean ± SD (*n* = 3). ***p ≤ 0.001, *p ≤ 0.05, ns: nonsignificant (I) Western blot indicating increase in the level of p‐ERK1/2 protein in CML patients with deletion of FUBP3 gene (Del1 and 2) as compared to patients without deletion (Non‐Del 1 and 2). GAPDH was used as a loading control (J) Increase in the level of p‐ERK1/2 protein in patients with deletion of FUBP3 gene (Del1 and 2) as compared to patients without deletion (Non‐Del 1 and 2) after normalization with loading control GAPDH using densitometric analysis.

Since the transcriptomic analysis of FUBP3 knockdown cells showed enrichment of the MAPK pathway, we evaluated the level of the proteins from this pathway, post FUBP3 knockdown. Among all the mediators of the MAPK pathway, we found that FUBP3 knockdown led to a significant increase in the level of *p*‐ERK1/2 protein (Figure 5A,B). *p*‐p38 and *p*‐JNK protein levels showed no significant difference upon FUBP3 knockdown (Figure S8A–D).

To further investigate if ERK is an effector of FUBP3, studies were conducted wherein 10 μM PD184352; a MEK/ERK kinase inhibitor, was used to inhibit the ERK signalling pathway. The increase in *p*‐ERK1/2 levels in FUBP3 knockdown cells was drastically reduced upon treatment of these cells with PD184352 (Figure 5C). To further confirm the regulation of ERK signalling by FUBP3 protein, cell survival and clonogenic assays were performed. As shown above, the CFU assay again showed a significant increase in the number of colonies formed post FUBP3 knockdown, however, treatment of these cells with PD184352 significantly led to a reduction (*p* ≤ 0.001) in colony numbers, indicating a decrease in proliferation and reversal in phenotype due to ERK inhibition (Figure 5D,E). Considering imatinib is the first line of therapy in CML, K562 cells were also treated under suspension and clonogenic culture conditions with their respective IC50 dose of 0.5 μM and 0.2 μM imatinib (Figure S9A,B). Cell survival assay showed a significant increase in the survival of FUBP3 knockdown cells post 0.5 μM imatinib treatment (*p* ≤ 0.05) (Figure 5F). However, a combined treatment of FUBP3 knockdown cells with 0.5 μM imatinib and 10 μM PD184352 significantly abrogated the gain in survival in these cells (*p* ≤ 0.001) (Figure 5F). The treatment of FUBP3 knockdown cells with 0.2 μM imatinib under clonogenic conditions also resulted in the formation of a significantly higher number of colonies, indicating lesser sensitivity of these cells to imatinib treatment as compared to control and scrambled cells (*p* ≤ 0.01) (Figure 5G). However, the incorporation of PD183352 resulted in a substantial reduction in the proliferation of these cells compared to imatinib treatment alone (*p* ≤ 0.001) (Figure 5G). Similar observations were also made with next‐generation TKI, 0.6 nM dasatinib, and 40 nM bosutinib, wherein increased survival of FUBP3 knockdown cells post TKI treatment alone (*p* ≤ 0.05) was drastically subsided upon combinatorial PD184352 treatment (*p* ≤ 0.001) (Figure 5H). The effect of FUBP3 on ERK expression was also investigated in patient samples with and without FUBP3 deletion, wherein we observed that CML samples with FUBP3 deletion had increased levels of *p*‐ERK1/2 protein (Figure 5I‐J). The abrogation in the gain of phenotype in FUBP3 knockdown cells upon ERK inhibition substantially indicates that FUBP3 functions through downstream activation of ERK signalling.

### 3.5 FUBP3 regulates MAPK–ERK signalling through PRC2 complex via PAK1

**FIGURE 6:**
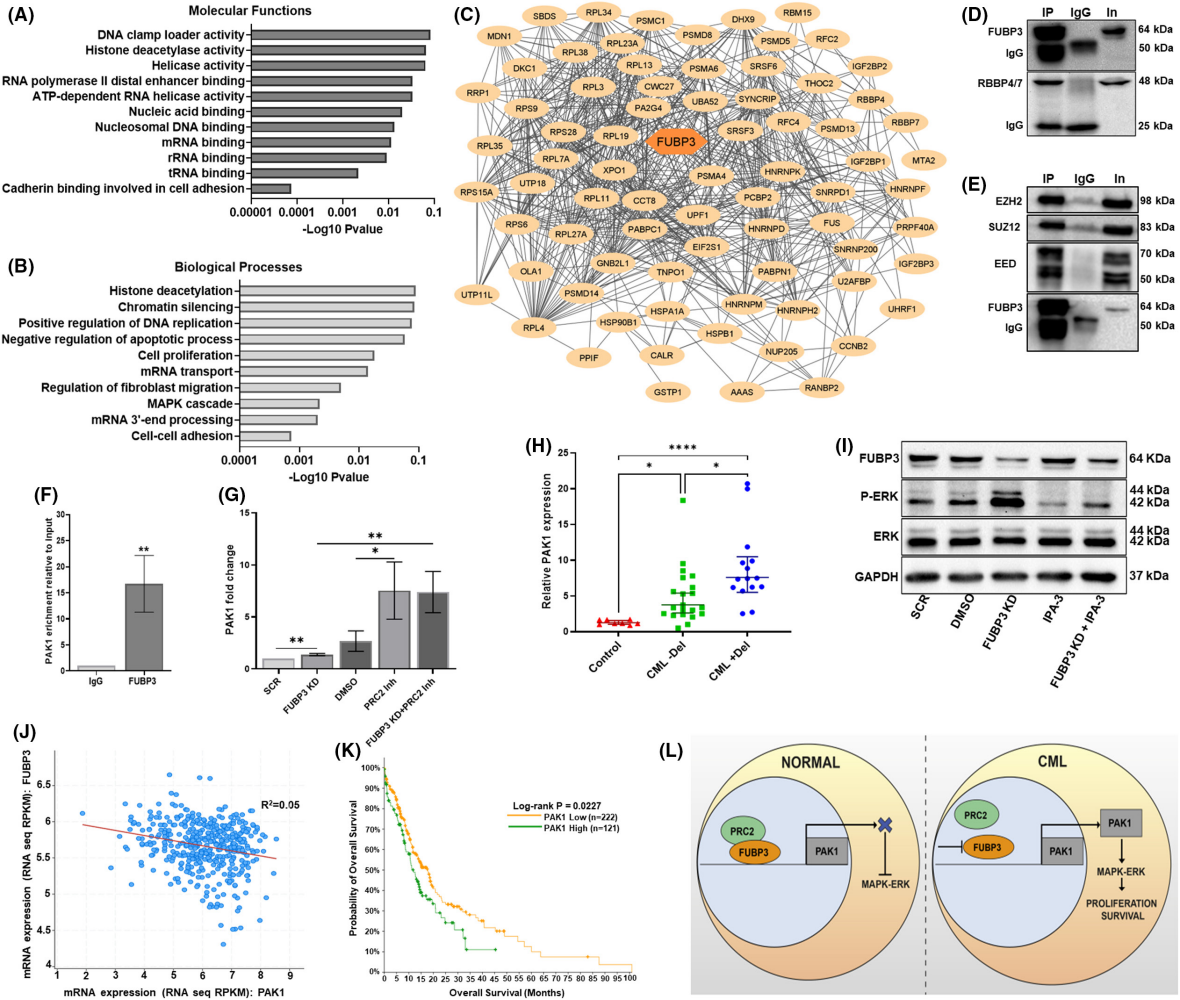
FUBP3 protein interacts with PRC2 complex to regulate MAPK–ERK signalling via PAK1 (A) GO analysis of biological processes of proteins interacting with FUBP3 enriched from FUBP3 pulldown mass spectrometry data with *p* < 0.05 and their corresponding ‐Log10 *p*‐value scores (B) GO analysis of molecular functions of proteins interacting with FUBP3 enriched from FUBP3 pulldown mass spectrometry data with *p* < 0.05 and their corresponding ‐Log10 *p*‐value scores (C) A protein network of FUBP3 interaction partners identified by mass spectrometric analysis of FUBP3 pulldown. The protein–protein network was generated using Cytoscape v3.9.0 (D) Representative image of western blot depicting the interaction of RBBP4/7 proteins in co‐immunoprecipitation of FUBP3 protein from K562 cells. L‐R: IP: FUBP3 pull‐down, IgG: equal amount of Mouse IgG, In: 10% input (E) Representative image of western blot depicting the interaction of SUZ12, EZH2 and EED proteins in co‐immunoprecipitation of FUBP3 protein from K562 cells. L‐R: IP: FUBP3 pull‐down, IgG: equal amount of Mouse IgG, In: 10% input (F) Enrichment of PAK1 DNA sequence relative to input in isotype IgG mouse and FUBP3 bound chromatin in K562 cells detected by RT‐PCR. Data are expressed as mean ± SD (*n* = 3) **: *p* ≤ 0.01 (G) The fold change difference in the relative expression of PAK1 gene under different conditions post‐treatment with PRC2 inhibitor; A‐395 determined by RT‐PCR. Data are expressed as mean ± SD (*n* = 3). SCR: scrambled siRNA, DMSO: (v/v) vehicle for A‐395, FUBP3 KD: FUBP3 siRNA, PRC2 Inh: PRC2 Inhibitor 12 nM A‐395. **: *p* ≤ 0.01, *p ≤ 0.05 (H) Relative mRNA expression of PAK1 gene in control samples and patients without (CML ‐Del) and with (CML + Del) deletion of FUBP3 gene detected by RT‐PCR. Each data point represents normalized mRNA expression. Horizontal lines represent median ± 95% class interval. ****p ≤ 0.0001, *p ≤ 0.05 (I) Representative image of western blot indicating the reduction in levels of phospho‐ERK1/2 protein in FUBP3 knockdown cells post‐treatment with PAK1 inhibitor; 12 μM IPA‐3. GAPDH was used as loading control. SCR: scrambled siRNA, DMSO (v/v) vehicle for IPA‐3, FUBP3 KD: FUBP3 siRNA (J) Negative correlation between expression of FUBP3 and PAK1 gene in leukaemia samples analysed by cBioPortal (Spearman's *r* = −0.18, *p* ≤ 0.01) (K) Kaplan–Meier survival curve showing the decrease in median overall survival (months) of patients with high (green) expression of PAK1 gene (11.97 months) as compared to those with low expression (orange) (17.66 months) (L) Schematic representation of the role of FUBP3‐PRC2 complex in CML progression under normal (left panel) and leukaemic conditions (right panel).

We have shown that FUBP3 knockdown results in ERK activation, which provides a proliferative advantage to these cells. To identify the transcriptional factors associated with FUBP3 mediated regulation of the ERK pathway, the FUBP3 protein was immunoprecipitated from a nuclear fraction (Figure S10A,B) and subjected to mass spectrometric analysis. The analysis resulted in 115 unique and 54 upregulated proteins in FUBP3 protein pulldown. GO analysis of mass spectrometric data identified biological processes (BP) and molecular functions (MF) involved in chromatin modification, splicing and stability of mRNA, regulation of cell proliferation, and adhesion (Figure 6A,B). The data also showed enrichment of UHRF1, RPL and FUS proteins previously reported to interact with FUBP3.^42^, ^43^, ^44^ A protein network of significantly enriched genes from the data is shown in Figure 6C. Interestingly, the data showed the interaction of retinoblastoma proteins RBBP4/7 (RBAP48/RBAP46) with FUBP3 protein. RBBP4/7 are highly homologous nuclear WD40 motif‐containing proteins facilitating the catalytic activity of the chromatin‐modifying PRC2 complex in vivo.^45^ Western blot analysis of the FUBP3 pulldown confirmed the interaction of FUBP3 with RBBP4/7 (Figure 6D) along with other members of the PRC2 complex; SUZ12, EZH2 and EED (Figure 6E).

Since members of the FUBP family are known to be transcriptional regulators,^46^ ChIP‐sequencing was performed to identify targets of the FUBP3 protein. A total of one hundred sixty–six peaks were identified. One of the gene sequences identified was p21‐activated kinase; PAK1. PAKs (PAK1‐6) are serine/threonine protein kinases known to activate the ERK pathway through MEK kinase.^47^ The binding site of the FUBP3 protein was 2099 bases away from the transcription start site within the intergenic region of the PAK1 gene. Upon validation by RT‐PCR, we observed ~16‐fold enrichment of PAK1 DNA sequence in FUBP3 bound chromatin compared to mouse IgG control (Figure 6F). PAK6, a homologous protein of PAK1, was shown to be epigenetically regulated by the PRC2 complex in hepatocellular carcinoma.^48^ However, the effect of the PRC2 complex on the expression of the PAK1 gene is still unknown. Interestingly, the inhibition of the PRC2 complex with 12 nM A‐395 (PRC2 inhibitor) resulted in a significant (~8‐fold) increase in PAK1 expression in FUBP3 knockdown cells (Figure 6G), suggesting that FUBP3 and PRC2 together regulate PAK1 expression. Furthermore, we observed a significant increase in PAK1 expression in CML patients with FUBP3 deletion compared to those without deletion (*p* ≤ 0.05) and control samples (*p* ≤ 0.0001) (Figure 6H). It has been previously reported that PAK1 regulates ERK signalling.^47^ Similarly, in the present context, inhibition of PAK1 using 12 μM IPA‐3 resulted in a decrease in *p*‐ERK1/2 levels, which were significantly upregulated in FUBP3 knockdown cells (Figure 6I). The cBioPortal analysis in acute leukaemias revealed a similar significant negative correlation between FUBP3 and PAK1 expression (*r* = −0.18, *p* = 0.001) (Figure 6J). Additionally, we also observed a significant decrease in median OS in patients with high (11.97 months) PAK1 expression as compared to those with low expression (17.66 months) (*p* = 0.0227) (Figure 6K). Collectively, the findings of this study imply that FUBP3 protein, together with PRC2 complex, regulates PAK1 expression that consecutively leads to an increase in ERK expression, which conduces towards disease progression and loss of response in CML (Figure 6L).

## 4 DISCUSSION

The differential response and development of resistance towards various generations of TKI has been a major impediment in the treatment of CML.^5^, ^6^ The present study establishes an association between genes near the ABL1‐BCR breakpoint on der (9) and their role in CML progression. We identified the microdeletions of ASS1, EXOSC2, FNBP1, FUBP3 and SMARCB1 genes in 8%–20% of CML patients. These findings are consistent with the previous studies.^10^, ^11^, ^12^, ^16^, ^17^ Among all the genes, the microdeletions of the FUBP3 gene only showed a significant association with poor TKI response and poor prognostic markers such as high Sokal score and increased total and platelet counts. A decrease in FUBP3 expression in CML patients was also associated with a higher percentage of myeloblasts and total counts and, most importantly, with poor TKI response outcomes. These findings establish the role of FUBP3 in influencing the response outcomes in CML patients. Also, there was a significant association of decreased FUBP3 expression with increased bone marrow fibrosis which is a central pathological feature in the monitoring of leukaemias, with progressive fibrosis being an indicator of adverse outcomes in CML patients.^49^ This observation was further supported by the upregulation of genes involved in fibrotic and inflammatory signalling in our transcriptomic data post FUBP3 knockdown. The data also revealed an increase in the expression of genes involved in TGF‐β, Wnt, and MAPK signalling cascades, which have been well established in the development of fibrosis.^50^, ^51^, ^52^ Though these observations have not been investigated in greater detail, it would be interesting to explore any alteration of these pathways in FUBP3‐mediated fibrosis.

We also observed an upregulation of the MAPK–ERK pathway post FUBP3 knockdown. The dysregulation of the MAPK–ERK signalling pathway is involved in the malignant transformation of several cancers,^53^ including leukaemia with ERK‐mediated increase in anti‐apoptotic proteins, BCL‐2, and BCL‐XL is contributing to disease progression.^54^ The decrease in FUBP3 expression also resulted in increased survival against the first and second generation TKI treatment and a higher number of colonies suggesting its role in self‐renewal and cell proliferation. The inhibition of ERK signalling abrogated the gain in phenotype in FUBP3 knockdown cells inferring the downstream regulation of ERK signalling by FUBP3 protein.

We further reported novel interactions of FUBP3 protein with SUZ12, EZH2, EED and RBBP4/7 proteins in this context. The members of the PRC2 family are known transcriptional and epigenetic regulators of genes involved in development and differentiation.^45^, ^55^ FUBP3 has been shown to interact with long noncoding RNA CMPK2 and drive colorectal cancer progression via activation of c‐Myc signalling.^56^ In this study, we have shown that PAK1 expression is regulated by FUBP3 protein. PAKs regulate several cell signalling pathways, including MAPK/ERK, p53, NFκB, SMAD and STAT3, involved in controlling tumour growth and survival.^57^ The combined inhibition of PRC2 and FUBP3 protein resulted in PAK1 overexpression, which in turn regulates ERK activation. The inhibition of PAK1 signalling by IPA‐3 along with imatinib has been shown to enhance the apoptosis of leukaemic cells^58^ inferring its role as a therapeutic target in CML.

This study is the first to suggest the tumour‐suppressive role of FUBP3 protein in CML and the association of microdeletions and decreased expression of the FUBP3 gene with adverse outcomes. The data provides a novel insight into the interaction of FUBP3 and PRC2 complex in the downstream regulation of ERK signalling via PAK1 (Figure 6L). Due to its oncogenic role in almost a third of cancers, the ERK pathway has been a focus of drug discovery for almost two decades, with Ras, Raf and MEK as the main targets.^59^ Further work needs to be done to identify the transcriptional regulators in FUBP3 signalling and the exact mechanism by which they modulate the target genes. It is imperative to incorporate novel markers such as FUBP3 for treatment evaluation in discerning and refining the treatment paradigm to beget better management of the disease. The findings also highlight the amalgamation of additional druggable targets for PAK1 and ERK signalling to the currently existing treatment regimens to improve clinical outcomes in CML patients.

## AUTHOR CONTRIBUTIONS

**Mugdha Sharma:** Conceptualization (equal); data curation (lead); formal analysis (lead); investigation (equal); methodology (equal); project administration (supporting); validation (lead); writing – original draft (equal); writing – review and editing (equal). **Seetharam Anandram:** Data curation (supporting); formal analysis (equal); investigation (equal); methodology (equal); validation (supporting); writing – original draft (equal); writing – review and editing (equal). **Cecil Ross:** Conceptualization (equal); data curation (supporting); formal analysis (equal); funding acquisition (lead); investigation (equal); methodology (equal); project administration (supporting); resources (lead); validation (supporting); writing – original draft (equal); writing – review and editing (equal). **Sweta Srivastava:** Conceptualization (lead); data curation (supporting); formal analysis (equal); funding acquisition (supporting); investigation (equal); methodology (equal); project administration (lead); resources (lead); validation (supporting); writing – original draft (equal); writing – review and editing (equal).

## CONFLICT OF INTEREST

The authors declare that they have no competing interests.

## Supporting information


Table S1

Table S2

Table S3

Table S4

Table S5

Figures S1–S10

## Data Availability

The datasets used and/or analysed during the current study are available from the corresponding author on reasonable request.
