# Supplementary material for: FUBP3 regulates chronic myeloid leukaemia progression through PRC2 complex regulated PAK1‐ERK signalling
Source: J Cell Mol Med. 2022 Dec 7;27(1):15–29. doi: 10.1111/jcmm.17584 (PMC9806296; doi:10.1111/jcmm.17584)
Supplement: Supplementary file 6 — Figures S1–S10 [file JCMM-27-15-s004.docx]

**Supplementary file**

**Figure S1.**

**(A)** Copy numbers of FUBP3 gene in CML patients with low and intermediate Hasford score. Each data point represents DNA copy number. Horizontal lines represent mean±SD. ns: non-significant

**(B)** Copy numbers of FUBP3 gene in CML patients with low and high EUTOS score. Each data point represents DNA copy number. Horizontal lines represent mean±SD. ns: non-significant

**Figure S2**

**(A)**

**(B)**

**(D)**

**(C)**

Relative mRNA expression of **(A)** EXOSC2 **(B)** ASS1 **(C)** FNBP1 **(D)** SMARCB1 genes in control and CML samples detected by RT-PCR. Each data point represents normalized mRNA expression. Horizontal lines represent median ± interquartile range. BM: bone marrow, B: blood. ** P<0.01, ns: non-significant

**Figure S3**

**
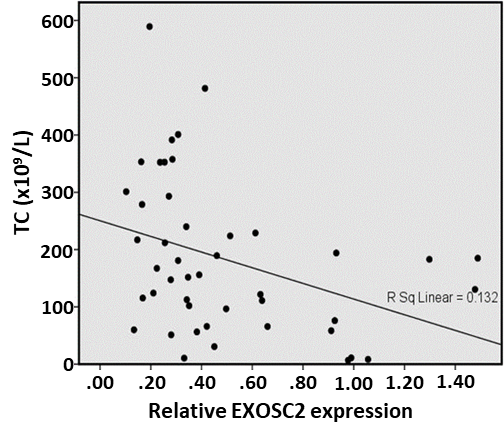
**

**(A)** Negative correlation between total counts (TC) (x10^9^/L) and relative mRNA expression of EXOSC2 gene in CML patients (Spearman’s r=-0.447, P<0.01)

**
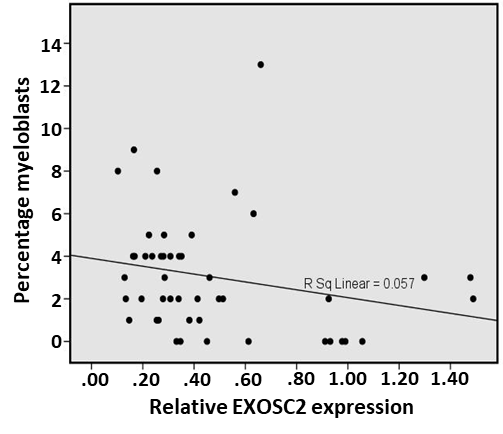
**

**(B)** Negative correlation between percent myeloblasts and relative mRNA expression of EXOSC2 gene in CML patients (Spearman’s r=-0.304, P<0.05)

**(C)** Scatter plot representing the expression of EXOSC2 gene in CML patients with optimal, warning, and failure TKI response. Each data point represents normalized mRNA expression. Horizontal lines represent median ±95% class interval

**Figure S4**

**(A)** Scatter plot representing copy numbers of FUBP3 gene validated in CML samples by TaqMan assay. The copy number value of 2 indicated normal values. The values close to 1 were considered deletions

**(B)** Graphical representation showing no off-target effect of FUBP3 siRNA on FUBP1 and FUBP2 expression. Bar graph representing the expression of FUBP1, FUBP2, and FUBP3 gene post FUBP3 siRNA treatment determined by RT-PCR. Data represented as mean ± SD (n=3)

**Figure S5**


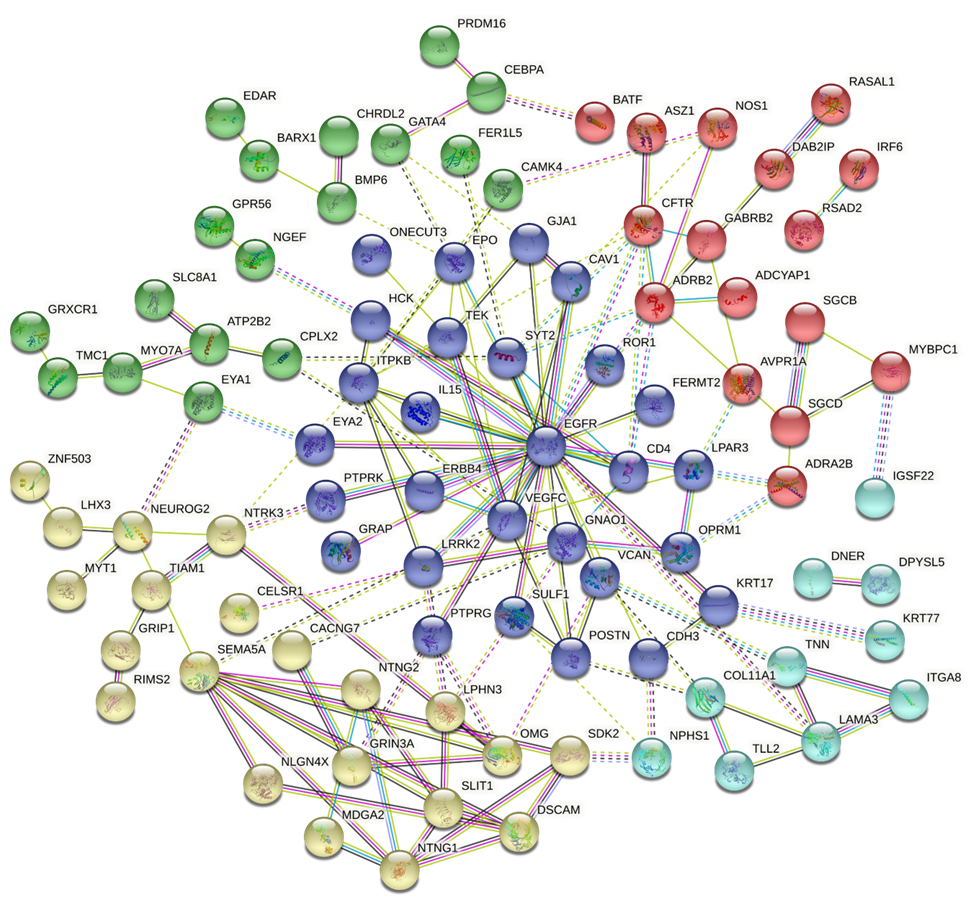


**(A)** A protein network of 124 downregulated genes enriched in FUBP3 knockdown cells generated using STRING. dB v11. Five clusters were identified using the k-means approach and are denoted by a separate color. The cluster showed enrichment of processes involved in the positive regulation of MAPK signaling, mRNA splicing, cell proliferation, and cell adhesion Each node represents a protein

**(B)** Bar graph representing the fold change difference in the expression of key downregulated genes from the STRING network generated using transcriptomic data in FUBP3 knockdown cells compared to scrambled control

**Figure S6**

1. Validation of transcriptomic data using RT-PCR. The expression of TAL2, HEY2, and TNS4 genes in scrambled and FUBP3 knockdown K562 cells. Data represented as mean ± SD (n=3)

1. Validation of transcriptomic data using RT-PCR. The increase in the expression of TFAP2B, NODAL, and PROX1 genes in FUBP3 knockdown cells over scrambled control. Data represented as mean ± SD (n=3)

**Figure S7**

1. Relative mRNA expression of BATF gene in control samples and patients without (CML -Del) and with (CML +Del) deletion of FUBP3 gene determined by RT-PCR. Each data point represents normalized mRNA expression. Horizontal lines represent median±95% class interval

1. Relative mRNA expression of HEY2 gene in control samples and patients without (CML -Del) and with (CML +Del) deletion of FUBP3 gene determined by RT-PCR. Each data point represents normalized mRNA expression. Horizontal lines represent median±95% class interval

1. Relative mRNA expression of TAL2 gene in control samples and patients without (CML -Del) and with (CML +Del) deletion of FUBP3 gene determined by RT-PCR. Each data point represents normalized mRNA expression. Horizontal lines represent median±95% class interval

**Figure S8**

**(B)**

**(A)**


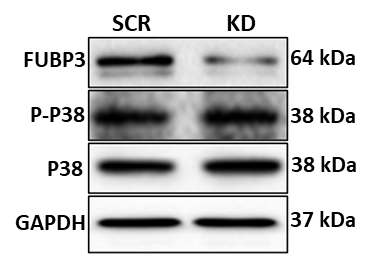


1. Representative image of western blot showing levels of FUBP3, phospho-p38, p-38, and loading control GAPDH protein in K562 cells treated under different conditions. L-R: SCR: scrambled siRNA, KD: FUBP3 siRNA. The same western blot for the FUBP3 knockdown experiment as represented in figure 5A was further probed for the expression of P-P38 and P38 proteins.
2. No significant fold change difference in the level of P-p38 protein was observed in FUBP3 knockdown cells compared to scrambled control. Data are represented as mean±SD (n=3) ns: non-significant


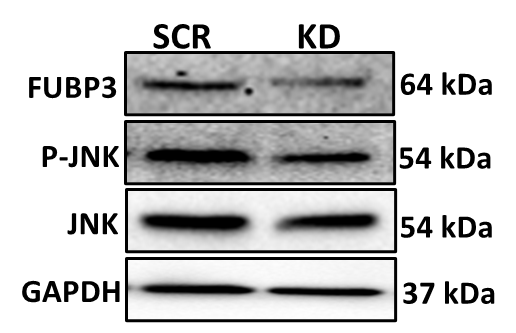


**(D)**

**(C)**

1. Representative image of western blot showing levels of FUBP3, phospho-JNK, JNK, and loading control GAPDH protein in K562 cells treated under different conditions. L-R: SCR: scrambled siRNA, KD: FUBP3 siRNA
2. No significant fold change difference in the level of P-JNK protein was observed in FUBP3 knockdown cells compared to scrambled control. Data are represented as mean±SD (n=3) ns: non-significant

**Figure S9**

1. Imatinib dose curve for K562 cells under clonogenic conditions. The scatter plot represents the number of colonies formed by K562 cells following treatment with various concentrations of imatinib in CFU assay. Data are represented as mean±SD (n=3)


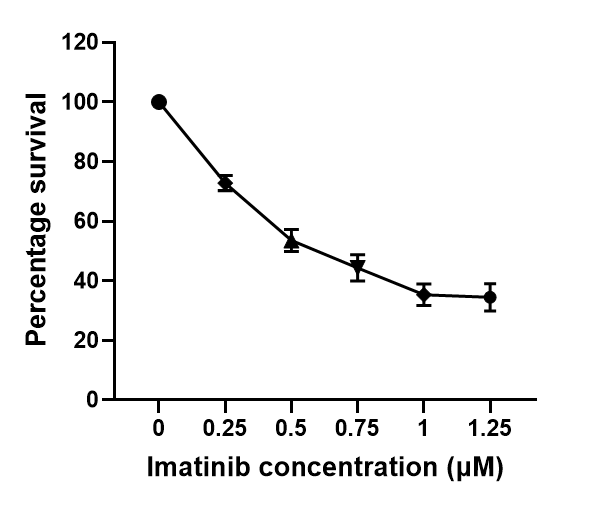


1. Imatinib dose curve for K562 cells under suspension conditions determined using WST-1 cell survival assay. The scatter plot represents cell viability as the percentage survival of K562 cells following treatment with various concentrations of imatinib. Data are represented as mean±SD (n=3)

**Figure S10**


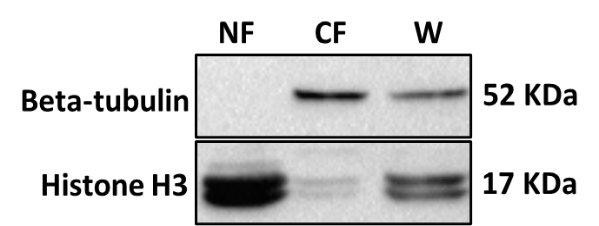


**(A)** The purity of subcellular fraction was assessed by western blotting against specific markers. The nuclear, cytosolic, and whole cell fractions are denoted by NF, CF, and W respectively. Nuclear marker: histone H3; Cytosolic marker: beta-tubulin


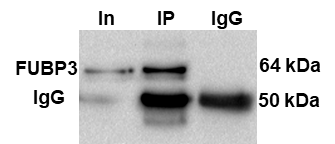


**(B)** Representative image of western blot depicting the immunoprecipitation of FUBP3 protein from the nuclear fraction of K562 cells. L-R: In: 10% input, IP: FUBP3 pull-down, IgG: equal amount of mouse IgG
